# Factors associated with herbal use among urban multiethnic primary care patients: a cross-sectional survey

**DOI:** 10.1186/1472-6882-4-18

**Published:** 2004-12-02

**Authors:** Grace M Kuo, Sarah T Hawley, L Todd Weiss, Rajesh Balkrishnan, Robert J Volk

**Affiliations:** 1 Department of Family and Community Medicine, Baylor College of Medicine, Houston, Texas, USA; 2 Division of General Medicine, University of Michigan and Ann Arbor VA Center for Practice Management and Outcomes Research, Ann Arbor, Michigan, USA; 3 University of Texas Health Science Center School of Public Health, Houston, Texas, USA

## Abstract

**Background:**

The use of herbal supplements in the United States has become increasingly popular. The prevalence of herbal use among primary care patients varies in previous studies; the pattern of herbal use among urban racially/ethnically diverse primary care patients has not been widely studied. The primary objectives of this study were to describe the use of herbs by ethnically diverse primary care patients in a large metropolitan area and to examine factors associated with such use. The secondary objective was to investigate perceptions about and patterns of herbal use.

**Methods:**

Data for a cross-sectional survey were collected at primary care practices affiliated with the Southern Primary-care Urban Research Network (SPUR-Net) in Houston, Texas, from September 2002 to March 2003. To participate in the study, patients had to be at least 18 years of age and visiting one of the SPUR-Net clinics for routine, nonacute care. Survey questions were available in both English and Spanish.

**Results:**

A total of 322 patients who had complete information on race/ethnicity were included in the analysis. Overall, 36% of the surveyed patients (n = 322) indicated use of herbs, with wide variability among ethnic groups: 50% of Hispanics, 50% of Asians, 41% of Whites, and 22% of African-Americans. Significant factors associated with an individual's herbal use were ethnicity other than African-American, having an immigrant family history, and reporting herbal use by other family members. About 40% of survey respondents believed that taking prescription medications and herbal medicines together was more effective than taking either alone. One-third of herbal users reported using herbs on a daily basis. More Whites (67%) disclosed their herbal use to their health-care providers than did African-Americans (45%), Hispanics (31%), or Asians (31%).

**Conclusions:**

Racial/ethnic differences in herbal use were apparent among this sample of urban multiethnic adult primary care patients. Associated factors of herbal use were non-African-American ethnicity, immigrant family history, and herbal use among family members. Whereas Hispanics and Asians reported the highest rates of herbal use, they were the least likely to disclose their use to health-care professionals. These findings are important for ensuring medication safety in primary care practices.

## Background

The use of complementary and alternative medicine (CAM) in the United States gained greater popularity in the 1990s. Two national telephone surveys of 1,539 and 2,005 adults, respectively, demonstrated an increasing trend in the use of CAM, including relaxation techniques, herbal medicine, massage, chiropractic, and acupuncture[1,2]. Specifically, the use of these unconventional treatments rose from 33.8% in 1990 to 42.1% in 1997. These surveys found that use of herbal medicine within the past year increased from 2.5% in 1990 to 12.1% in 1997[2]. CAM use was also found to be more frequent among females, persons 35 to 49 years of age, persons of ethnicities other than African-American, persons who were college educated, and persons whose annual income was greater than $50,000[2]. In a separate study also conducted in the 1990s, the American Botanical Council estimated that one-third of the nation's adults use herbal remedies[3].

Efficacy studies of herbal supplements are on the rise, but most data published to date are preliminary and do not provide strong evidence for the clinical effectiveness of herbs. Nevertheless, about 15 million American adults (18%) are thought to use prescription medications concurrently with herbal or vitamin products[4], and as many as 70% of persons who use herbal remedies do not discuss their use of such remedies with their physicians or pharmacists[1,5-7]. By not communicating about herbal use, they may put themselves at increased risk for adverse drug-herb interactions[8] and make it extremely difficult for health-care professionals to monitor them for such interactions[9]. Likewise, patients do not know what symptoms they should report to their health-care provider that indicate potential adverse effects of drug-herb interactions. Consequently, unintentional medication errors could occur.

The prevalence of herbal use among racially/ethnically diverse primary care patients varies from study to study[2,3,5-7,10-12], ranging from 30%[5,6] to 77%[7]. Since patients must interact with their primary care providers and pharmacists for illnesses to be diagnosed and quality medical care to be provided, a better understanding of variations in herbal use patterns among primary care patients is needed. To this end, we conducted a study with two objectives: 1) to describe the herbal use of ethnically diverse patients in a large metropolitan area and to examine factors associated with herbal use; and 2) to investigate perceptions about and patterns of herbal use among those patients.

## Methods

### Setting and study population

We implemented this cross-sectional study within the Southern Primary-care Urban Research Network (SPUR-Net) from September 2002 to March 2003. SPUR-Net is a practice-based research network in Houston, Texas, that consists of five constituent member organizations affiliated with a county health system, a managed care organization, or a private practice clinic. SPUR-Net clinicians provide care to patients from diverse ethnic and socioeconomic backgrounds, with approximately one million patient visits per year. A total of six primary care clinics were included in this study that varied according to socioeconomic status (SES) of their patients as measured by income level and insurance type. For the purposes of this study, we defined "clinic SES" according to the insurance status of the majority of patients; "high SES" means that most patients have insurance (i.e., private insurance and/or Medicare), and "low SES" means most patients are indigent (i.e., county health-care coverage and/or Medicaid). Human subject approvals were obtained from the Institutional Review Boards at all of the SPUR-Net constituent organizations. Permission to conduct the study was also obtained from the medical directors and applicable patient advisory groups at each of the six participating clinics.

To be eligible for participation in the study, patients had to be at least 18 years of age and to be visiting one of the participating clinics for routine, nonacute care. A target of 50 surveys in each of the six clinics was collected from a convenience sample of patients. The decision regarding the number of patients to be surveyed was limited by our resources, including availability in funding and personnel. A research assistant approached potential subjects in the clinic setting to determine their willingness to complete a 23-item questionnaire about herbal use in either English or Spanish. Those patients who consented to participate were either given the survey to complete on their own or had the survey administered to them by the research assistant. Research assistants were available on-site to answer any questions the patients had, helping to improve patients' understanding of the terms used in the survey. Recruitment methods were the same in all of the participating clinics. The research assistants stopped recruiting patients when a minimum of 50 surveys was collected in each clinic.

### Survey instrument

Survey questions were adopted and modified from previously developed and validated surveys on CAM use, including national telephone surveys conducted by Eisenberg et al.[1,2,13], a family practice survey by Elder et al.[5], a research clinic survey by Johnson et al.[3], and a national mail survey by Astin et al[14]. We modified these questions for use among our multiethnic patient population; we also translated the survey questions into Spanish. The survey instrument was pilot tested with 54 English-speaking subjects and 10 Spanish-speaking subjects before the study. The survey was reviewed by several groups of patient representatives in the community health centers to ensure consistency in responses. For example, some members of a patient advisory group representing a homeless clinic perceived herbal use to be marijuana use; for this reason, we decided not to include this patient population in our study.

The final survey instrument had three components. First, all participating patients answered questions regarding sociodemographic characteristics (e.g., gender, age, race, ethnicity, education, immigrant family history, herbal use by other family members, spoken language other than English, and clinic location). Immigrant family history and spoken language were elicited with the following questions: "Are your family members immigrants to the United States (Y/N)?," and "Do you speak another language other than English?" After completing the demographic questions, respondents answered a series of questions regarding their belief in herbal use and their herbal information sources. The questions pertained to their personal use of herbs (Y/N); their belief in the benefit of herbal remedies (Y/N), the source of their herbal information (physician, pharmacist, family, friends, etc.); their preferred content of herbal information (e.g., effectiveness, side-effects, interactions with other medications), and their preferred methods for obtaining herbal information from physicians or pharmacists (e.g., handout, World Wide Web site, consultation). Patients who reported using herbal supplements answered additional questions related to their patterns of and reasons for herbal use. In open-ended questions, the participating patients were asked about the herbs they specifically used and the health conditions for which they took the herbal products. Related questions included frequency of herbal use (daily, frequently-few times/month, occasionally—few times/year); duration of use (< 1 year, 1–2 years, 3–5 years, > 5 years); expenditure on herbal products; reported concomitant use of prescription medications; disclosure of herbal use to physicians or pharmacists; and any experiences of adverse reactions from using herbs.

For the purposes of this study, we used the definition of dietary supplements stipulated in the 1994 Dietary Supplement and Health Education Act (DSHEA) to differentiate herbs from vitamins and minerals. Herbal use was defined as having ever used herbal products or natural medicines for health maintenance or treatment of health conditions. To measure herbal use, we asked the following question: "Do you use any of the following?" Response options included: herbs/herbal products or natural medicine (e.g., echinacea, St. John's wort, ginseng, ginkgo biloba, soy supplements), folk medicine or home remedy, vitamins, minerals, or none. Herbal use did not include the use of folk medicine, home remedies (such as honey), vitamins, or minerals.

### Data analysis

Data from the paper-based survey were entered into an ACCESS database and were imported into SAS 9.1 for Windows. The study variables were summarized by using one-way frequencies to examine the sociodemographic characteristics of the study sample, the belief in and information source for herbal use, and the patterns of and reasons for herbal use among urban multiethnic primary care patients. The frequencies of use of specific herbs were counted, and the health conditions for which herbs were used were further coded into three types—acute, chronic, and health maintenance.

Based on findings from previous studies, we used the following independent variables as reference variables for both the univariate and multivariate logistic regression analyses: male gender, age less than 30 years, African-American ethnicity, less than a college education, no immigrant family history, no herbal use by other family members, and visiting a high SES clinic. A Chi-square test of proportions was used to determine the association between herbal use and each of the independent variables related to demographic characteristics; a *p *value ≤ 0.05 was considered to be statistically significant. In order to assess factors associated with herbal use, all hypothesized variables (age, gender, race and ethnicity, education, immigrant family history, herbal use by other family members, and clinic clientele stratified by SES) were included in both the univariate and the multivariate logistic regression analyses. These independent variables were entered as dichotomous variables in the model: gender (male vs. female), age (< 50 years, ≥ 50 years), ethnicity (African-American vs. other, including Whites and Hispanics), education (less than college vs. college and greater), immigrant family history (yes vs. no), herbal use by family members (yes vs. no), and clinic clientele (high SES vs. low SES). Significant variables identified by backward elimination of the main effects from the multivariate analysis were further evaluated in two-way interactions. Thus, the final model contained all of the significant main effects and the two-way interaction terms. Odds ratios and 95% confidence intervals were calculated to determine the effects of the significant variables on herbal use. Since the sample size for Asians was small, Asians were not included in the logistic regression analyses. Furthermore, the language variable was excluded from the regression analyses because the survey question was not clearly answered by many patients; for example, 10 Spanish-language forms had "no language other than English" indicated. In addition, some answers were possibly indicative of an exclusive language other than English instead of the bilingual capability of the respondent.

## Results

### Description of sample

Of the 327 patients who agreed to participate in the survey, only 322 completed the race/ethnicity information and were included in the analysis. The characteristics of the study sample are summarized in Table 1. Two-thirds of the patients were female, and approximately half of all the patients had less than a college education. More than a third (37%) of the patients reported having an immigrant family history, and 50 patients (15%) used the Spanish-language form to complete the survey.

**Table 1:** Descriptive Characteristics of the Study Sample (n = 322)

Variables	White n (%)	Hispanic n (%)	African American n (%)	Asian n (%)
Totals	68(21.1)	98(30.4)	136(42.2)	20(6.2)
Gender				
Male	20(29.4)	34(34.7)	37(27.4)	7(35.0)
Female	48(70.6)	64(65.3)	98(72.6)	13(65.0)
Age (yrs)				
< 30	13(19.1)	17(17.3)	27(19.8)	4(20.0)
30–49	34(50.0)	34(34.7)	47(34.6)	5(25.0)
50+	21(30.9)	47(48.0)	62(45.6)	11(55.0)
Education				
< High School	3(4.4)	52(53.0)	17(12.6)	0
High School	16(23.5)	23(23.5)	54(40.0)	5(25.0)
≥ College	49(72.1)	23(23.5)	64(47.4)	15(75.0)
Immigrant Family History				
No	57(86.4)	41(41.8)	101(77.1)	0
Yes	9(13.6)	57(58.2)	30(22.9)	20(100.0)
Herbal Use by Other Family Members				
No	43(63.2)	42(42.9)	95(69.9)	11(55)
Yes	25(36.8)	56(57.1)	41(30.1)	9(45)
Clinic Type				
High SES Clinic	40(58.8)	14(14.3)	52(38.2)	9(45)
Low SES Clinic	28(41.2)	84(85.7)	84(61.8)	11(55)

### Herbal use

Overall, 36% of our study sample reported ever using herbs. The proportions of herbal users varied across racial/ethnic groups, with use being reported by 50% of Hispanics, 50% of Asians, 41% of Whites, and 22% of African-Americans. Herbal use by other family members was reported to be 41% (57% among Hispanics, 45% among Asians, 37% among Whites, and 30% among African-Americans). Patients who reported using herbs indicated that they received information about those herbs mainly from family members and relatives. Nevertheless, most patients reported that they preferred receiving herbal information (e.g., on effectiveness, side-effects, and drug interactions) through handouts or brochures from their physicians or pharmacists, followed by having access to a consultation service or a Web site. About 40% of all of the survey respondents, but especially Asians (55%) and Whites (47%), believed that taking prescription medications and herbal medicines together was more effective than taking either alone. About 41% of Hispanic respondents believed that herbal medicines were superior to prescription medications, as compared to 12% of Whites. These differences in beliefs about herbal use among the ethnic groups were found to be statistically significant (*p *< 0.05). Nearly half of the patients who reported using herbs (46%), particularly Hispanics (63%) and Asians (57%), also reported taking prescription medications concomitantly with the herbs (Table 2). Since our survey question was designed to measure self-reported concomitant herbal use and prescription drug use, we cannot confirm whether or not those who reported taking both were actually using both.

**Table 2:** Patterns of and Reasons for Herbal Use Among Urban Multiethnic Primary Care Patients (n = 322)

Variables	White n (%)	Hispanic n (%)	African American n (%)	Asian n (%)
Herbal Use	28(41.2)	49(50.0)	30(22.1)	10(50.0)
Daily Herbal Use*	14(48.3)	13(22.8)	13(33.3)	4(30.8)
Herbal Use 3+ Years*	12(41.1)	45(78.9)	17(45.9)	7(53.8)
Report Taking Herbs and Prescription Medications for the Same Health Problems*				
	10(33.3)	36(63.2)	17(32.7)	8(57.1)
Told Physicians/Pharmacists About Herbal Use*				
	20(66.7)	17(30.9)	21(44.7)	4(30.8)
Had a Bad Reaction*	2(7.4)	1(2.0)	3(11.1)	0
Believed Both Prescription Medications and Herbal Medicines Are Better Than Either Alone**				
Agree	32(47.1)	28(28.6)	54(40.6)	11(55.0)
Disagree	14(20.6)	44(44.9)	48(36.1)	6(30.0)
Neither	22(32.4)	26(26.5)	31(23.3)	3(15)
Believed Herbal Medicines Are Superior to Prescription Medications***				
Agree	8(12.3)	40(41.2)	31(23.1)	6(30.0)
Disagree	36(55.4)	20(20.6)	62(46.3)	8(40.0)
Neither	21(32.3)	37(38.1)	41(30.6)	6(30.0)
Received Herbal Information (multiple)				
Family or relatives	20(29.4)	60(61.2)	43(31.6)	10(50.0)
Magazines	24(35.3)	19(19.4)	38(27.9)	5(25.0)
Television	13(19.1)	24(24.5)	45(33.1)	0
Internet	12(17.7)	7(7.1)	9(6.6)	4(20)
Physician	10(14.7)	8(8.2)	12(8.8)	2(10.0)
Pharmacist	2(2.9)	2(2.0)	6(4.4)	0
Preferred Herbal Information (multiple)				
Effectiveness	53(77.9)	72(73.5)	87(64.0)	9(45.0)
Side-effects	42(61.8)	76(77.6)	82(60.3)	12(60.0)
Interactions	46(67.7)	67(68.4)	75(55.2)	9(45.0)
Preferred Method for Obtaining Herbal Information (multiple)				
Handout/Brochure	45(66.2)	80(81.6)	84(61.8)	11(55.0)
Website	25(36.8)	11(11.2)	20(14.7)	6(30.0)
Consultation	29(42.7)	20(20.4)	47(34.6)	5(25.0)

*Indicates only those patients who reported herbal use

**p = 0.008;*** *p *< 0.0001

### Factors associated with herbal use

Variables demonstrating a significant univariate association (*p *< 0.05) with herbal use were ethnicities other than African-American, immigrant family history, and herbal use by other family members (Table 3). In the multivariate logistic regression model, non-African-American race/ethnicity (OR = 2.42, 95% CI, 1.33–4.40), immigrant family history (OR = 2.23, 95% CI, 1.20–4.14), and reported herbal use by other family members (OR = 7.98, 95% CI, 4.48–14.18) remained significant predictors of reported herbal use (*p *< 0.05). In addition, interactions between immigrant family history and herbal use by other family members were found to be significant terms in the model (Table 3). With the race/ethnicity variable adjusted, having an immigrant family history was associated with a 19 times greater likelihood of herbal use among those whose family members also use herbs. When the analyses were run with the Asian group included, the results did not change.

**Table 3:** Univariate Analysis of Factors Associated with Herbal Use Among Urban Multiethnic Primary Care Patients (n = 302)

Variables	Total	Herbal Use n (%)	X^2 ^*p*-value
Gender			
Male	91	32(35.2)	0.9
Female	210	75(35.7)	
Age (yrs)			
< 30	106	34(32.1)	0.4
≥ 30	196	73(37.2)	
Race/Ethnicity			
African-American	136	30(22.1)	<0.0001
White & Hispanic	166	77(46.4)	
Education			
< College	165	57(34.6)	0.8
≥ College	136	49(36.0)	
Immigrant Family History			
No	199	56(28.1)	0.0001
Yes	96	49(51.0)	
Herbal Use by Other Family Members			
No	180	31(17.2)	<0.0001
Yes	122	76(62.3)	
Clinic Type			
High SES Clinic	106	35(33.0)	0.5
Low SES Clinic	196	72(36.7)	

### Perceptions about and patterns of herbal use

The reasons given by the study subjects for herbal use included faster resolution of symptoms (47%), the desire to try alternative therapies (33%), and preference for having their own methods to care for their health (20%). Among the herbal users, 32% reported taking herbs on a daily basis, and 60% reported using herbs for longer than three years. Usage varied by race/ethnicity; for example, 48% of Whites reported taking herbs on a daily basis, and 79% of Hispanics reported using herbs for longer than three years.

Even though Hispanics and Asians used herbs more frequently, they were the least likely to disclose their herbal use to their physicians or pharmacists. More Whites (67%) told their health-care professionals about their herbal use than did the African-Americans (45%), Hispanics (31%), or Asians (31%). The reasons given for nondisclosure generally fell into two main categories: 1) "They (the provider) never asked," and 2) "It wasn't important for them to know." While few respondents (5.3%) reported having experienced an adverse reaction to herbs, many of them (43%) did not inform their physicians of it.

The specific herbs used by the patients covered a wide spectrum and varied by ethnicity. The herbs used most commonly by White patients were echinacea (32.1%), St. John's wort (21.4%), ginkgo biloba (14.3%), and chamomile (14.3%). Hispanic patients most often reported using chamomile (61.2%), aloe vera (44.9%), and garlic (20.4%). African-American patients reported primarily using garlic (40%), ginseng (30%), and ginkgo biloba (10%). The herbs used by Asian patients were garlic (50%), ginkgo biloba (30%), and ginger (30%). Other herbs that were reported by patients—albeit infrequently—included Yun Zhi, black cohosh, dong quai, guggle phosphate, bee pollen, cat claws, and "a shot of whiskey." The patients who reported using herbs used them for a wide range of health problems, such as boosting the immune system, improving memory, and treating insomnia, depression, or diabetes. For conditions considered to be chronic, 44% of the White patients reported herbal use versus 32% of African-American patients. For conditions considered to be acute, 71% of Hispanic patients used herbs versus 10% of Asians. For health maintenance, 50% of Asian patients used herbs versus 16% of Hispanic patients.

## Discussion

Our data show that herbal use is common (36%) among urban multiethnic primary care patients, but has a wide variability among racial/ethnic groups. Hispanics and Asians reported the highest rates of herbal use (50%), and African Americans reported the lowest (22%). Previous research conducted in the western United States found that the prevalence of herbal use among racially/ethnically diverse primary care patients varies[2,3,5-7,10-12], ranging from 30% among primary care patients residing in urban settings on the west coast of the United States[5,6] to 77% among primary care patients residing in the largest United States—Mexico border city[7].

As expected, factors associated with herbal use included race/ethnicity, having an immigrant family history, and herbal use by other family members. In addition, we found interactions between having an immigrant family history and herbal use by other family members. Previous studies did not examine such interactions and found age to be predictive of herbal use[2,6,7]. Unlike other investigators, we did not find a significant relationship between age and herbal use. Other investigators, however, did not account for interactions such as those addressed in our analysis. One study (n = 113) found no significant differences in the use of CAM therapies that could be attributable to gender, educational level, age, race, or clinic attended[5]. Another study (n = 542) found an association between the use of CAM therapies, high education level, and female gender[6]. In addition, a recent study conducted in a large United States—Mexico border city revealed that 77% of the residents surveyed (n = 547) use all modalities of CAM therapies and that such use was associated with a high education level[7]. When the residents reported specifically using herbal and home remedies (59%), however, herbal use was found to be associated with a low education level[7].

We found that nearly half of herbal users (46%) reported taking herbal medicines and prescription medications concomitantly. More importantly, 43% of herbal users reported not disclosing their herbal use to their physicians or pharmacists. Interestingly, Hispanics and Asians used herbs the most frequently but disclosed their herbal use to their physicians or pharmacists less often than did Whites and African Americans. This lack of communication about herbal use is an area of concern because of the potential for medication errors and untoward reactions to herb-drug interactions. Adverse drug-herb interactions pose a great danger for patients. For example, ginkgo biloba, garlic, and ginseng all may interact with Coumadin^® ^(warfarin sodium) and cause an increase in bleeding time[15,16]. Echinacea, an immunostimulant, can counteract the action of the immunosuppressants (e.g., the corticosteroids prednisone, methotrexate, and cyclosporine) used to treat immune disorders[17,18]. The interaction between St. John's wort and cyclosporine—which is used to treat rheumatoid arthritis and psoriasis and to prevent the rejection of a transplanted organ—could result in decreased availability of cyclosporine and, consequently, to the worsening of arthritis or psoriasis or the rejection of a transplanted organ [19-23]. St. John's wort may also interact with antidepressants, such as monoamine oxidase inhibitors (e.g., Nardil^®^, Parnate^®^) and potentiate the effects of selective serotonin reuptake inhibitors (e.g., Paxil^®^, Prozac^®^, Zoloft^®^)[24]. Moreover, drug-herb interactions might adversely affect the monitoring of certain drug therapies and might even cause life-threatening complications. For example, ginseng, hawthorn, licorice, kyushin, plantain, and uzara root have the potential to interfere with the monitoring of Lanoxin^® ^(digoxin)[25]. In addition, kava has been associated with hepatitis[26] and has resulted in coma when used with Xanax^® ^(alprazolam)[27]. As these detrimental effects have been realized, concern about the increased use of herbal supplements has grown[2,28-33].

Two-thirds of the patients we surveyed reported wanting to receive information on herbal medicines from their physicians or pharmacists, preferably in the form of a handout or a brochure. These findings suggest that future studies are warranted to develop and test educational materials to 1) deepen our understanding of racial/ethnic variation in herbal use among primary care patients; 2) educate health-care professionals about the variations in the use patterns and the rationales for use that may help to reduce medication errors and increase the quality and safety of medical care; and 3) educate patients regarding evidence-based herbal information and encourage patients to communicate their herbal use to their physicians/pharmacists.

Our study results should be interpreted in the context of several limitations. First, our estimates of herbal use frequency are imprecise because we used a convenience sample instead of identifying patients by randomized sampling. Secondly, even though we adopted the DSHEA definition of herbs, some patients had difficulty understanding this definition. Specifically, a small group of patients thought that herbs were equivalent to prescription medications such as digoxin and aspirin; the patients' level of understanding of herbs was improved after the research assistants provided further explanation and clarification. Third, we discovered that asking questions, such as "What do you take when you run out of your medications?," was more effective in eliciting answers from the study subjects than when asking them, "Do you use herbs, herbal products or natural medicine?." For these reasons, we had research assistants on-site to help facilitate the survey process. Fourth, the patients surveyed reported their concomitant use of herbs with prescribed medications based on their perceptions and memories. Last, we did not include measures of quality of life or questions about patient satisfaction with herbal use, which would be helpful in future studies, especially when comparing multiethnic and socioeconomically diverse patient groups.

## Conclusions

Despite these limitations, our findings confirm the increasing frequency of herbal use as reported in previous studies. Our study also gives a unique perspective by focusing on factors associated with reported herbal use among an urban multiethnic primary care patient population. In particular, we found that patients with immigrant family history—especially those with family members who use herbs—are most likely to report herbal use. Perhaps most disconcerting was our finding that while an increasing number of primary care patients report taking herbal medicines concomitantly with prescription medications, many of them do not disclose their herbal use to their physicians or pharmacists. These findings suggest that primary care clinicians need to understand the extent and patterns of herbal use by their multiethnic patients and efforts to elicit information from patients about herbal use may be warranted. Future studies are needed to develop effective interventions for primary care health-care professionals and patients to improve medication safety by eliminating potential adverse herb-drug interactions and medication errors.

## Competing interests

The author(s) declare that they have no competing interests.

## Authors' contributions

GMK conceived of the study, designed the survey questionnaires, coordinated and managed the data-collection process, directed data analysis, and drafted the manuscript. STH participated in drafting the manuscript and helped with data analysis. LTW performed the statistical analysis and participated in drafting the manuscript. RB helped with data analysis. RJV reviewed the questionnaires and data analysis, and participated in drafting the manuscript. All authors read and approved the final manuscript.

**Table 4:** Multiple Logistic Regression Analysis of Factors Associated with Herbal Use Among Urban Multiethnic Primary Care Patients (n = 302)

Variable	OR	95% CI
***Main Effects***		
Gender		
Male	1.00	
Female	1.12	(0.60–2.11)
Age (yrs)		
< 30	1.00	
≥ 30	1.37	(0.74–2.55)
Race/Ethnicity		
African-American	1.00	
White & Hispanic	2.42*	(1.33–4.40)
Education		
< College	1.00	
≥ College	1.11	(0.58–2.15)
Immigrant Family History		
No	1.00	
Yes	2.23*	(1.20–4.14)
Herbal Use by Other Family Member		
No	1.00	
Yes	7.98*	(4.48–14.18)
Clinic Type		
High SES Clinic	1.00	
Low SES Clinic	0.80	(0.40–1.60)
***Interactions***		
Immigrant Family History * Herbal Use by Other Family Members		
	19.39	(8.11–46.38)

*p < 0.05

## Pre-publication history

The pre-publication history for this paper can be accessed here:


